# ALS and Frontotemporal Dysfunction: A Review

**DOI:** 10.1155/2012/806306

**Published:** 2012-08-07

**Authors:** Eugene Y. Achi, Stacy A. Rudnicki

**Affiliations:** Department of Neurology, University of Arkansas for Medical Sciences, Little Rock, AR 72205, USA

## Abstract

Though once believed to be a disease that was limited to the motor system, it is now apparent that amyotrophic lateral sclerosis (ALS) may be associated with cognitive changes in some patients. Changes are consistent with frontotemporal dysfunction, and may range from mild abnormalities only recognized with formal neuropsychological testing, to profound frontotemporal dementia (FTD). Executive function, behavior, and language are the most likely areas to be involved. Screening helpful in detecting abnormalities includes verbal or categorical fluency, behavioral inventories filled out by the caregiver, and evaluation for the presence of depression and pseudobulbar affect. Patients with cognitive dysfunction have shortened survival and may be less compliant with recommendations regarding use of feeding tubes and noninvasive ventilation. Evolving knowledge of genetic and pathological links between ALS and FTD has allowed us to better understand the overlapping spectrum of ALS and FTD.

## 1. Introduction

Amyotrophic lateral sclerosis, or ALS, was first described by Charcot in the nineteenth century. Much of his clinical description continues to hold true to this day. Patients experience progressive, painless weakness that may originate in the arm, leg, or bulbar musculature. Associated with this is atrophy of the muscles; fasciculations may also be seen. In addition to these lower motor neuron manifestations, upper motor neuron findings are found on examination including increased tone, exaggerated deep tendon reflexes, and pathological reflexes such as a Babinski sign or jaw jerk. Sensation and bowel and bladder function are typically spared [1]. In Charcot's description, cognitive changes were not described. Marie, a peer of Charcot's, described emotional lability in patients with ALS in 1892 [2], and reports of ALS patients with cognitive changes including, irritability, delusions, and hallucinations, date to at least the early part of the twentieth century [3–6]. However, for many years it remained entrenched in the teaching of neurology that the body wasted yet the mind was spared in ALS. This may for a while have been a self-fulfilling prophecy; since it was not expected, it was not looked for by physicians and not reported by families. The very nature of the symptoms related to ALS may have also created obstacles to recognizing cognitive symptoms; for example, ALS patients may stop working because of their weakness or slurred speech, so may not be in a setting requiring them to perform complex planning and decision making. The concept of clinics devoted to the care of ALS patients, pioneered by Stan Appel and Forbes Norris, means that ALS patients, once seen at most a few times by their local neurologist and then cared for by an internist until their death, are now being seen regularly throughout the course of their disease by neurologists who were seeing tens of hundreds of ALS patients rather than one or two per year. As reports of patients with motor neuron disease (MND) associated with dementia were published in increasing numbers [7–18] and the clinical features of frontotemporal dementia were better described including consensus criteria first published in 1998 [19, 20], it became clear that the dementia seen in ALS patients is best characterized as FTD [21, 22]. This review will address what constitutes frontotemporal dysfunction in ALS and how frequently it occurs, how to best evaluate cognition in ALS patients, and what is understood about the pathology and genetics of ALS and FTD.

## 2. Frontotemporal Dementia 

FTD has an insidious onset with a slowly progressive course with age of onset typically in the 50's and 60's; hence early on it was referred to as presenile dementia. There is relative preservation of memory, praxis, and visuospatial skills with impairment of behavior, language, and/or personality. Patients characteristically lack insight into their problems. Initial features at presentations may include changes in behavior (behavioral variant or bvFTD), difficulty with expression of language but with relative preservation of comprehension (primary progressive aphasia or nonfluent progressive aphasia), or impaired language characterized by anomia in conjunction with impaired comprehension (semantic dementia) [23]. Patients with bvFTLD may be disinhibited, apathetic, or manifest stereotypical behaviors (Table 1) [23, 24]. Features associated with nonfluent progressive aphasia may include anomia, phonemic paraphasia, grammatical errors, stuttering, oral apraxia, alexia, or agraphia [20, 25, 26]. Semantic dementia, the least common type of FTLD, is characterized by speech that is fluent and grammatically correct but empty of content. Naming of people, both familiar and famous, is frequently impaired, and while confrontational naming is very poor, repetition is generally preserved [27]. Executive dysfunction is common early in FTD; when seen in Alzheimer's disease it typically occurs later [19, 28]. Executive dysfunction is reflected in problems with planning, organizing, abstracting, and prioritizing, along with impaired verbal fluency [19–22].

## 3. Frontotemporal Dementia and ALS

In one of the earlier reviews of dementia and ALS, Hudson identified 60 families with ALS, including 9 (15%) families who also had dementia. In addition, he reported 42 sporadic cases of ALS with either dementia or parkinsonism, suggesting overlap between various neurodegenerative syndromes. [29] Autopsies in early reports of patients with both ALS and dementia found minimal to marked frontotemporal atrophy but absence of Alzheimer's changes with no granulovacuolar degeneration or tangles [7, 29].

Patients with ALS-FTD typically have onset of symptoms in their 50's, and like ALS without dementia, it is slightly more common in men than women. The ALS symptoms may precede, occur simultaneously, or follow the signs and symptoms of FTD, though the most common finding is to have cognitive change first followed by weakness. Interval between the cognitive symptoms and weakness may be a few months to up to 7 years, with a mean of 2 years [7, 29–35]. Some [30–32, 36] but not all [37, 38] authors found bulbar onset disease more often in patients with ALS-FTD compared to those with ALS alone. At least some patients may have significant upper extremity weakness while lower extremities are relatively preserved, with maintained ambulation even at the time of death [7, 8, 29, 33, 36, 39]. Behavioral changes may include euphoria, indifference, and personality changes, while language impairment includes paucity of speech, echolalia, impaired comprehension, and even mutism [7, 29, 36, 39].

Survival in patients with ALS-FTD is worse compared to those with ALS alone or FTD alone [30, 40]. In those who are cognitively impaired but not frankly demented, the type of frontotemporal dysfunction may influence survival. ALS patients with dysexecutive function may have worse survival but those with abnormalities limited to language or visuospatial skills have similar survival compared to the cognitively normal ALS patient [40, 41]. ALS patients with dementia primarily characterized by poor memory such as seen in Alzheimer's disease, did not have further shortening of survival [37, 40].

EEGs are frequently normal though may show background or focal slowing [8, 9, 42]. SPECT scan may show reduced uptake in the frontal lobes [8, 39, 42]. In a voxel-based morphometry study, MRI's of both ALS and ALS-FTD patients had atrophy in the frontotemporal regions compared to controls, though frontal atrophy was greater in the ALS-FTD patients [43]. 

Strong and colleagues proposed the following classification system for the frontotemporal syndromes in ALS: ALSci or cognitively impaired, ALSbi or behaviorally impaired, ALS-FTD in which Neary criteria for FTD are met, FTD-MND like in which patients clinically had FTD and pathologically have motor neuron loss but did not manifest signs of motor neuron disease during life, and ALS-dementia in which patients have Alzheimer's or vascular dementia. Patients with ALSci may have impaired verbal fluency or executive dysfunction, and those with ALSbi have the behavioral features associated with frontotemporal dysfunction, but do not meet Neary criteria for FTD [44]. Patients with the behaviorally predominant form of ALS-FTD may also have impaired language, with reduced verbal fluency as the most common language deficit found [21, 45]. Occasional ALS-FTD patients have primary progressive aphasia as the main manifestation of their dementing illness [46, 47]. Many of these patients become mute as their disease progresses [36, 48]. For those with profound dysarthria secondary to weakness of bulbar musculature, it may be difficult to recognize the degree of their aphasia. However, if they have sufficient hand strength to hold a pen, the degree of language impairment may be apparent through their writing.

Overall, memory is relatively preserved in patients with ALS-FTD, and the memory problems reported are believed by memory problems reported are believed by most neurologists to be related to frontal dysfunction. Patients may have difficulty with retrieving memories and have poor learning strategies [38]. Lack of concentration and poor attention may also play a role in a patient's performance [49]. In keeping with the dementia of ALS not fitting an Alzheimer's-like pattern, the Mini-Mental Status Examination is typically normal in ALS patients [32].

## 4. Cognitive Testing in Patients with ALS

Following ever increasing papers reporting frank dementia with ALS, investigators started to pursue whether more subtle cognitive changes in patients may be present in some patients. Testing is potentially problematic; slurred speech makes it difficult to do verbal testing, hand weakness may mean written tests are not possible, and timed tests need to be adjusted to accommodate slowness related to the ALS. However, as many patients are now followed in multi-disciplinary ALS clinics at regular intervals, some clues of early cognitive changes may come to light in this setting. For example, patients who previously had no problems understanding the instructions on how to do a vital capacity may have difficulty figuring out how to time their breaths for the test as their ALS progresses. Those who communicate by writing and early on had perfect syntax and spelling may start to make spelling and grammatical errors later on in their disease. Such observations piqued the interest of investigators to determine the frequency and extent of cognitive problems in the ALS patient.

In an attempt to minimize the limitations dysarthria and hand weakness create for testing in this population, a neuropsychological battery administered by computer touch screen, the Cambridge Neuropsychological Test Automated Battery (CANTAB), was used to study 69 patients with ALS. In addition to performing the CANTAB, treating neurologists were asked to determine if they believed the patient was demented based upon standard criteria for dementia, and based upon FTD specific criteria for dementia. Using traditional criteria, 7% of patients were classified as demented, and 33% possibly demented. Using FTLD criteria, the number of patients classified as demented jumped to 22%, while 27% where classified as possibly demented. Patients clinically diagnosed with dementia had difficulty with new learning and planning, along with rigid thinking and slowness of processing information in keeping with frontotemporal dysfunction [50]. 

Using a caregiver questionnaire avoids the limitations posed by the patient's physical disabilities. In addition, because patients with FTD frequently are oblivious to behavioral changes, caregivers can better identify them. The Neuropsychiatric Inventory (NPI) [51], Frontal Behavioral Inventory (FBI) [52], Cambridge Behavioral Inventory (CBI) [53], and Frontal Systems Behavioral Scale (FrSBe) [54] were designed by the cognitive neurologists to help screen for frontotemporal dysfunction. The FrSBe has both a self-rating as well as a caregiver form; the NPI, CBI, and FBI are all caregiver-only forms [51–54]. Having used both the NPI and CBI in our patient population, we found the latter more useful in identifying behavioral changes [55, 56]. Apathy is identified in up to 56% of patients in studies using CBI or FrSBe [57–59] and correlated with reduced verbal fluency [60] but not with arterial blood gasses or the ALS functional rating scale [58]. Besides apathy, other common findings from using one of these caregiver inventories have included stereotypical behaviors in 20% [61], executive dysfunction in 34–48% [41, 59], and disinhibition in 18–27% [41, 61]. Using the CBI, 11% of the patients met criteria for FTD [61]. There have been conflicting results regarding correlating degree of abnormalities found with bulbar onset disease [59, 61]. Depression was found in up to a third of the caregivers; it was related to the patient's behavioral symptoms in one study [62] but not confirmed in a subsequent study [61]. 

Lomen-Hoerth and colleagues screened 100 patients with ALS for cognitive abnormalities using verbal fluency and the Mini-Mental Status Examination (MMSE). Thirty-one had abnormal verbal or categorical fluency in one minute, defined as a score of fewer than 8 “d” words and less than 13 animals. If they were anarthric, they could write their answers. Half of the patients with bulbar onset disease had an abnormal score while approximately a quarter of patients with limb onset disease had an abnormal score. No patient had an abnormal score on the MMSE. All 100 patients were asked to undergo more extensive testing, 44 agreed and had additional tests of executive function, memory, language, and visuospatial skills. Of these, 18 had results consistent with probable or definite FTD, with 12 having the primarily behavioral type, 4 with primary progressive aphasia, and 2 with semantic dementia. An additional 5 patients had possible FTD. FTD was more likely to be diagnosed in older patients, those with a family history of dementia, Parkinson's disease, or ALS, and had a lower forced vital capacity [32].

Of 160 patients studied with a cognitive battery that evaluated executive function, memory, language, and visuospatial skills, 14% met Neary criteria for FTD, 21% had executive dysfunction, 14% were impaired in one of the other areas tested, and 47% were cognitively normal. Patients with executive dysfunction were older and had more rapid disease progression, but there was no relationship to having bulbar onset ALS [63]. In another study of 279 patients, similar distributions were found; 15% had FTD, 49% were cognitively normal, 32% were mildly impaired, and 13% moderately impaired. Areas most likely to show abnormalities included attention, concentration, and working memory; confrontational naming and memory were also abnormal, though to a lesser degree [64].

 In a study of 40 patients, the diagnosis of dementia required memory dysfunction, as well as impairment in at least 2 other domains (executive function, language, visuospatial skills, and attention). Using this definition, 70% of the patients were cognitively normal, 13% had probable, 10% had possible dementia, and 8% were mildly cognitively impaired. The larger number of cognitively normal subjects reported in this series compared to others is likely a reflection that memory impairment had to be present, while this is usually missing in FTD. Similar to other studies, impairment of executive function including letter and category fluency was the most likely test to be abnormal. There was no association with cognitive function and bulbar onset, respiratory status, or disease duration [37].

There are a relative paucity of studies that have looked at cognitive function in ALS patients over time; those that have been done have showed varying results. In one such study of 52 patients retested every 4 months, while verbal and nonverbal fluency and concept formation were abnormal compared to controls, they did not decline significantly over time [65].

In two small studies with patients undergoing repeat testing at 6 months in one and 9 months in the other, there was no significant decline on the whole [66, 67]. However, at both test times, ALS patients performed in the abnormal range in generating “s” words. At the second test, ALS patients scored at least 1 standard deviation below the scores of controls on the following tests: verbal and written word generation, motor free visual perception test, and recognition memory test of faces. Overall, bulbar onset patients did less well than limb onset patients on cognitive testing, and their performance worsened on repeat evaluation [66]. As individuals, 7/19 patients declined in tests over time, but in only one performance was abnormal for the majority of tests [67].

 What about the patients with FTD? How often do they show signs of motor neuron disease or frank ALS? Thirty-six patients with FTD had a detailed neuromuscular examination and nerve conduction studies and electromyogram (EMG). According to the El Escorial criteria, 5 patients had definite ALS and one patient had neurogenic changes limited to a single limb. Six patients had dysphagia of unclear cause. Five patients had fasciculations but EMG revealed no other abnormalities. However, one of these patients went on to develop definite ALS a year later [68]. In a retrospective study of patients who first presented with behavioral variant FTD and were followed until their death, 18 of 61 subsequently developed ALS. Those who developed ALS were more likely to have an onset of symptoms of delusions and word finding difficulties and less likely to have memory problems compared to those that purely had FTD [30]. A study of 319 FTD patients with extended followup found that motor neuron disease only developed in 8 of them [69].

Primary lateral sclerosis (PLS) and progressive muscular atrophy (PMA) are viewed as ALS variants, the former associated with purely upper motor neuron dysfunction and the latter with purely lower motor neuron involvement. Neuropsychological testing in patients with PLS has found mild cognitive impairment tests of frontal function including verbal associative fluency and psychomotor speed [70]. Autopsy of 2 cases of FTD (one with executive dysfunction followed by aphasia, the other with expressive aphasia and apraxia) found TDP-43 and ubiquitin positive inclusions in frontal and temporal neurons as well as neuronal loss in the motor cortex and degeneration in corticospinal tracts. There was no evidence for lower motor neuron pathology. One patient had been diagnosed with PLS premorbidly; the other had Parkinsonism but was not seen for 4 years prior to death [71]. A review of 5 cases of possible FTD-PLS identified only one patient whose autopsy found no lower motor neuron involvement [72]. A minority (17%) of patients with PMA had cognitive impairment defined as 2 standard deviations below the norm in letter-number sequencing and immediate and delayed story recall [73]. Overall, there is a sense that patients with PMA and PLS are more likely to be cognitively normal compared to those with ALS though further testing is warranted.

## 5. Screening for Cognitive Impairment in the Clinic

Extensive neuropsychological testing in every patient seen in the ALS clinic is not feasible for most clinics. It can be expensive, time consuming, and for patients with marked weakness and dysarthria, not technically realistic. Therefore, developing a screening battery that can be done within these limitations is important. When testing ALS patients, one also needs to take into account respiratory status, pseudobulbar affect, medications, depression, and pain. In a 2009 practice parameter update from the American Academy of Neurology, the authors acknowledged lack of consensus on how to best study cognitive changes in patients with ALS [74]. There is currently an FTD task force as part of the Northeast ALS (NEALS) Consortium working on this, as well as an effort by the NIH to identify common data elements for investigators to use, including studying cognition in ALS patients [75]. Several investigators have developed screening tools to evaluate cognition in ALS patients. The ALS Cognitive Behavioral Screen, developed by Wooley and colleagues, includes a 15-item ALS specific behavioral questionnaire filled out by the caregiver, and an 8-item cognitive assessment of the patient that is estimated to take only 5 minutes; it has been validated [76]. The Penn State Screen Battery of Frontal and Temporal Dysfunction Syndromes takes approximately 20 minutes; and is currently being used in a nationwide study to establish its validity [77, 78]. The UCSF screen battery is the longest of the screens, taking approximately 45 minutes to complete. It includes an ALS specific version of the FBI, written verbal fluency, the ALS Cognitive Behavioral Screen, an emotional lability scale, and the Beck Depression Inventory-II [75]. In my clinic, I screen with category fluency, the CBI, and antisaccade testing [55, 79]. The latter consists of asking the patient to look in the opposite direction to your wiggling finger, and is a measure of frontal lobe inhibition [80]. For patients whose bulbar dysfunction precludes spoken tests of fluency, written verbal fluency can be performed. Patients are given five minutes to write “s” words and four minutes to write “c” words that contain only four letters; an index is calculated taking into account how long it takes the patient to copy the same words they spontaneously wrote [81].

## 6. Pathology of ALS-FTD

Patients with FTD pathologically may demonstrate a tauopathy; in those without a tauopathy, the pathology was originally described as FTD-U for ubiquitin positive but tau-negative inclusions [82]. In 2006, 2 groups reported that the ubiquitinated inclusions seen in FTD with ALS were TAR-DNA-binding protein 43 or TDP-43 [83, 84]. Burden and distribution of the TDP-43 varied depending upon the clinical phenotype. Patient with pure ALS have TDP-43 pathology primarily in the spinal cord, those with pure FTD have TDP-43 pathology primarily in the cortex, while those with FTD-ALS have TDP-43 pathology in both areas [85, 86]. However, TDP-43 inclusions are not only found in the expected areas for ALS and FTD, but can also be seen in the cerebellum, parietal, and even occipital lobes although to lesser degrees, suggesting TDP-43 is part of a multisystem neurodegenerative process [87]. TDP-43 levels in CSF are higher in patients with ALS or FTD, but there is overlap between the patient groups as well as with controls [88].

In a patient with ALS, FDT, and Parkinsonism, TDP-43 pathology was found not only in the motor system, hippocampus, and amygdala; it was also prominent in the globus pallidus, caudate, and putamen [89]. This spectrum of TDP-43 burden that reflects clinical manifestations give further proof that ALS and FTD may share similar mechanisms of protein misfolding.

SOD-1 mutations are responsible for approximately 20% of the familial cases of ALS [90]. 

Although ubiquitin positive neuronal inclusions were found at autopsy of patients with SOD1-associated ALS, the inclusions were not immunoreactive with TDP-43 [91]. 

## 7. The Genetics of ALS and FTD

The link between ALS and FTD has been further forged as we have learned more about the genetics of both diseases. The majority of ALS is sporadic, but 5–10% is inherited. The first gene identified as playing a role in familial ALS was the superoxide dismutase gene, or SOD1, and is believed to be responsible for about 20% of the familial ALS cases; mutations are also found in approximately 2% of sporadic ALS [90, 92]. There is no association between the SOD1 gene and dementia; patients with the SOD1 mutation compared to those with sporadic ALS performed better on cognitive testing and at a level similar to controls [93]. FTD has a higher familial incidence than ALS; with 42–45% of FTD patients having a positive family history for a similar disorder [94, 95]. Within the same families there were cases of ALS, FTD; and ALS-FTD identified. In 2000, a genome wide-linkage analysis of two large data sets consisting of over seven hundred families, found a genetic locus between D9S301 and D9S167 on chromosome 9q21-q22 linked to ALS with FTD [96]. A previously identified senataxin or SETX gene mutation, also located on chromosome 9 (9q34), has been linked to the rare juvenile-onset dominant FALS, however, the two loci above do not overlap, and the pattern of the disease is very different in each [97]. A second genome-wide linkage study in a large ALS/FTD kindred in 2006 also showed linkage to chromosome 9, but at 9p13.3-21.2 [98]; similar results were found by other researchers as well [99, 100]. In 2011, two groups of researchers were able to uncover the specific mutation within this locus leading to ALS/FTD. It was an expansion of a GGGGCC hexanucleotide repeat in the intron of protein *C90RF72* leading to an alternative splicing of this protein that is the responsible mutation [101, 102]. Less than 23 repeats corresponded with a wild-type allele, while more than 30 repeats corresponded with individuals expressing the disease. The US-based study found a prevalence of this expansion in 12% of familial FTD and 22.5% of FALS [101]. The study involving the European population, found higher prevalence rates: 46% in familial ALS, 21% in sporadic ALS, and 29% in familial FTD [102]. Clinically, the patients differ from the typical patient with FTD because of prominent psychiatric symptoms, including delusions, paranoia, and irrational thoughts [103]. While TDP-43 deposition is found in patients with this mutation, p62 positive cytoplasmic inclusions in the hippocampus and cerebellum are another hallmark pathological feature [104, 105]. 

Discovery that TDP-43 accumulated in neurons in both ALS and FTD [83, 84] led to sequencing of the TARDBP gene in ALS patients. Mutations in the gene were found in 1–3% of patients with both sporadic and familial ALS and patients with the mutations had TDP-43 pathology at autopsy [92, 104–107]. 

A recent study in Nature involving 19 individuals within a five-generation family uncovered a new gene, UBQLN2 mutation underlying X-linked dominant inheritance of ALS/FTD. This mutation led to ubiquilin 2 protein pathology in the spinal cord and brain. Functional analysis showed an impaired degradation of ubiquinated proteins, a well-known function of the ubiquilin 2 proteins [108]. 

Motor neuron disease associated with FTD and Parkinsonism, that is, disinhibition-dementia-parkinsonism-amyotrophy complex (DDPAC), is a rare entity that has been linked to a microtubule-associated protein tau gene mutation (MAPT) on chromosome 17q21. This disease is a separate entity from the ALS/parkinsonism dementia complex specific to the Guam population, and pathologically is characterized by a large amount of cytoplasmic accumulation of filamentous tau inclusions [109, 110]. Tau stabilizes and conducts transport of vesicles along cellular microtubules, and neurodegenerative disease has been theoretically linked to these cytoplasmic tau inclusions. 

## 8. Conclusions

When cognitive change was first discussed at ALS meetings, it was met with some skepticism; over time, it has become well accepted that there may be a spectrum of cognitive changes in patients with ALS [74, 86, 111]. Some patients have normal cognition throughout their disease; others have mild cognitive impairment, while relatively few are frankly demented. Abnormalities identified are typically ones that imply involvement of the frontotemporal lobes, including dysexecutive syndrome, behavioral changes, and language dysfunction. The frequency and severity of cognitive changes in ALS has varied widely in reports to date, likely in part related to the tests used and how dementia was defined [29–35, 37, 38]. The challenges of trying to evaluate cognition in patients who may have trouble speaking and writing cannot be ignored. Does it matter that patients with ALS may not be cognitively intact? It is true that some patients would rather not know that cognition is affected, and this may influence the number of patients who are willing to enroll in studies investigating this topic. Family members, however, may be reassured when they learn some of the things they have noticed in their loved one can be explained by cognitive decline that is part of the disease process. It may also influence treatment and equipment needs. For example, a patient with cognitive impairment may not be able to learn to use a computer-based augmentative language device. Patients who lack insight into the severity of their disease may not understand the importance of trying to adapt to using noninvasive ventilation, or may not be appropriately cautious when walking with leg weakness. Patients with ALS-FTD were less likely to be compliant with recommendations for percutaneous endoscopic gastrostomy or use of non-invasive ventilation compared to cognitively intact patients [41, 112]. Patients with ALS may have difficulty interpreting the emotions associated with facial expressions, even when they are otherwise cognitively normal; this may impact their relationships with their caregiver and possibly influence medical decision making [113]. Caregivers of those with behavioral symptoms have lower quality of life, higher caregiver burden, and higher rates of depression [59]. Behavioral problems may need to be managed; the selective serotonin uptake inhibitors have been found to be beneficial in treating patients with FTD, and so likely should be tried in the ALS patients who have behavioral management issues as well [114].

## Figures and Tables

**Table 1:** Behavioral features in FTLD [23, 24].

Disinhibited type
Increased interest in sexual activity
Lack of judgment
Swearing
Violation of personal space
Impulsive buying
Paranoia
Criminal activity
Grandiose thinking
Ignoring social etiquette
Apathetic type
Blunted emotions
Disinterested and withdrawn
Lack of attention to personal hygiene
Lack of empathy
Stereotypical type
Hoarding
Food fads, overeating
Ritualistic/repetitive behavior
